# The antidepressant roles of Wnt2 and Wnt3 in stress-induced depression-like behaviors

**DOI:** 10.1038/tp.2016.122

**Published:** 2016-09-13

**Authors:** W-J Zhou, N Xu, L Kong, S-C Sun, X-F Xu, M-Z Jia, Y Wang, Z-Y Chen

**Affiliations:** 1 Department of Neurobiology, Shandong Provincial Key Laboratory of Mental Disorders, CAS Center for Excellence in Brain Science and Intelligence Technology, School of Medicine, Shandong University, Jinan, Shandong, China; 2 Department of Clinical Laboratory, Second Hospital of Shandong University, Jinan, Shandong, China

## Abstract

Wnts-related signaling pathways have been reported to play roles in the pathogenesis of stress-induced depression-like behaviors. However, there is relatively few direct evidence to indicate the effect of Wnt ligands on this process. Here, we investigated the role of Wnts in mediating chronic restraint stress (CRS)-induced depression-like behaviors. We found that CRS induced a significant decrease in the expression of Wnt2 and Wnt3 in the ventral hippocampus (VH) but not in the dorsal hippocampus. Knocking down Wnt2 or Wnt3 in the VH led to impaired Wnt/β-catenin signaling, neurogenesis deficits and depression-like behaviors. In contrast, overexpression of Wnt2 or Wnt3 reversed CRS-induced depression-like behaviors. Moreover, Wnt2 and Wnt3 activated cAMP response element-binding protein (CREB) and there was CREB-dependent positive feedback between Wnt2 and Wnt3. Finally, fluoxetine treatment increased Wnt2 and Wnt3 levels in the VH and knocking down Wnt2 or Wnt3 abolished the antidepressant effect of fluoxetine. Taken together, our study indicates essential roles for Wnt2 and Wnt3 in CRS-induced depression-like behaviors and antidepressant.

## Introduction

Major depression is a widespread psychiatric disorder with very high prevalence rate up to 15% in the world.^1^ Over the past decade, a number of studies have found mechanisms underlying depression and available antidepressants according. For example, glycogen synthase kinase 3 (GSK3) has been shown to participate in the pathogenesis and treatment of major depression disorders.^2, 3, 4, 5^ Lithium, a well-used mood stabilizer for both bipolar and depression, can inhibit the activity of GSK3β.^6^

Wnt pathway plays essential roles during embryogenesis, nervous system development and adult hippocampal neurogenesis (AHN).^7, 8, 9^ Wnts are secreted glycoproteins that activate different signaling pathways, including canonical Wnt/β-catenin pathway, non-canonical planar cell polarity (PCP) pathway and Wnt/Ca^2+^ pathway. GSK3 is primarily regulated by Wnt,^10^ and interestingly, it has been shown that Wnt/β-catenin signaling participates in depression disorders.^11, 12, 13^ The activation of Wnt/β-catenin signaling promotes antidepressant effect, whereas the blockade of Wnt/β-catenin signaling increases depression behaviors.^3, 14, 15, 16, 17, 18^

Chronic stress is a risk factor for developing psychiatric diseases, such as major depression and anxiety disorders.^19^ Increasing evidence shows that some Wnt-related signaling molecules are altered under stress condition. For example, chronic unpredictable stress decreases the expression of Frizzled 6 (Fzd6), a Wnt receptor.^20^ The expression of dishevelled-2 (DVL2), a key molecule in the Wnt signaling cascade, is also downregulated in the nucleus accumbens (NAc) subjected to chronic social defeat stress.^21^ Acute and chronic stress increases the expression of Dickkopf-1 (Dkk-1), an endogenous inhibitor of Wnt/β-catenin signaling, in the hippocampus.^22^ However, there is few evidence to indicate the effect of Wnt ligands on the process of stress. Although previous microarray studies of the hippocampus have identified decreases in the Wnt2 transcript after chronic restraint stress (CRS),^23^ no direct results could verify the relationships between Wnts and stress-induced depression behaviors. In the present study, we have used CRS in mice as a useful animal model of depression and aimed to investigate whether selective Wnts participated in stress-induced depression-like behaviors.

## Materials and methods

### Animals

Adult male C57BL/6 mice (8 weeks old) were kept in cages in a 12 h light/dark cycle with food and water available unless otherwise noted (Vital River Laboratories, Beijing, China). Three to four mice were housed in one cage. The room temperature was maintained at 20±2 °C. All procedures were performed in accordance with the National Institutes of Health Guide for the Care and Use of Laboratory Animals and were approved by the institutional animal care and use committee of Shandong University.

### Chronic restraint stress

Mice were assigned randomly into the CRS group or the no CRS (No-CRS) group. The CRS group was restrained during the morning (0800-1000 hours) daily for 2 h for 14 days in well-ventilated polypropylene restrainers without food and water. After CRS treatment, mice were back to the home cage. Mice were weighed daily before the restraint stress treatment. In some series of experiments (Figure 5), stressed mice were once-daily administered with saline or fluoxetine (10 mg kg^−1^, Sigma Aldrich, St Louis, MO, USA) i.p.

### Brain area microdissection

Brains were removed and placed in a mouse brain slicer (Braintree Scientific, Braintree, MA, USA). The dorsal hippocampus (DH) was defined as anteroposterior (AP): −0.94 to −2.30 mm and the ventral hippocampus (VH) as AP: −2.46 to −3.80 mm.^24, 25^ Coronal sections (1-mm thick) were collected and the DH and VH were isolated under the dissecting microscope following delineations from the mouse brain atlas.^24, 25, 26^ Then the tissues were kept at −80 °C until use. The exact separation of DH and VH was further confirmed by the expression of two specific genes lactose-phlorizinhydrolase (Lct) and Decorin (Dcn), which are primarily expressed in the DH and VH, respectively.^27, 28^

### Stereotaxic surgery and microinjection

Mice were anesthetized by an i.p. injection of 5% chloral hydrate (400 mg kg^−1^) and then placed in a stereotaxic apparatus. Mice received VH micro-infusions. The injection coordinates, relative to bregma, were as follows: AP, −2.8 mm; lateral (L),±2.5 mm; and dorsoventral (V), −2.3 mm. Lentiviruses (1 μl per side) were infused into the VH, and the infusion micro-syringe was then left for diffusion for an additional 2 min. The Wnt2 or Wnt3 siRNA sequence used for lentiviruses was as followed: Wnt2 siRNA antisense, 5′-GAGGTCATTTTTCGTTGGC-3′ Wnt3 siRNA antisense, 5′-TCGTAGATGCGAATACACT-3′. The siRNA plasmids and scramble plasmid used to package lentiviruses were purchased by Thermo Scientific Open Biosysterms (Waltham, MA, USA). The pUltra lentivirus vector was used to package Wnt2 or Wnt3-overexpression lentivirus with coexpressing a green-fluorescent protein (GFP). The promoter used in the overexpression lentiviral vector is the ubiquitin promoter and the promoter used in the siRNA lentiviral vector is the U6 promoter. The lentiviruses had no cell specificity.

### Behavioral procedures

The behavioral tests were administered between 0800 and 1600 hours. All behavioral tests were performed 24 h after the last restraint treatment. Different cohorts of male mice were used for these behavioral experiments. The sample sizes were chosen based on common practice in animal behavior experiments (7–10 animals per group). Investigators were not blinded to groups. The animals were allowed to habituate to the context before test. After the end of the behavioral research, mice that received a lentivirus microinjection were killed, and the GFP was detected via fluorescence microscopy to indicate the location of the virus. Animals were excluded from the analysis when the microinfusion sites were not correct.

### Sucrose preference test

The sucrose preference test (SPT) was measured as previously described.^29^ Mice were accustomed to sucrose solution (1%) for 2 days after the last day of CRS to avoid neophobia. On 16th day, 12 h after water deprivation, mice were given free access to two bottles filled with either 1% sucrose solution or water for 1 h. Then the weights of the bottles were measured and fluid consumption was determined. Sucrose preference was calculated as: sucrose preference (%)=sucrose intake/total fluid intake (sucrose+water) × 100.

### Forced swim test

The forced swim test (FST) was performed in a glass cylinder (25 cm in height and 18 cm in diameter, containing18 cm of water at 22 °C) as previously described, with some modifications.^30, 31^ Mice were forced to swim for 6 min, and the sessions were videotaped for later analysis by a blind observer. Immobility time during the last 4 min of the testing period was recorded. Immobility was defined as being stationary and floating in the water with minimal movements to keep the head above water. After the swim test, mice were dried and returned to their home cages.

### Tail suspension test

Tail suspension tests (TSTs) were performed using a procedure described previously, with slight modifications.^32^ The tail was wrapped with tape, leaving ~1 cm of the tail protruding. The long length of the tape was used to reduce tail-climbing. Mice were suspended by the tail for 6 min, and the test procedures were videotaped for analysis by a blind observer. Mice that climbed their tails were removed from the experimental analysis. Mobility was defined as movement of the hind legs.

### Hippocampal neuronal cultures and transfection

Cultured hippocampal neurons were prepared from newborn (P0) mice. Cultured neurons (7 DIV) were infected with lentivirus for 6 h. The medium was then replaced with fresh medium. CBP–CREB (cAMP response element-binding protein) interaction inhibitor was applied 3 days after lentivirus infection. Neurons were lysed 10 h after the administration of the CREB inhibitor.

### BrdU administration and immunohistochemistry

Mice were anesthetized with 5% chloral hydrate, perfused with 4% paraformaldehyde and the brains were postfixed overnight. Brains were cut into 30-μm-thick sections on a freezing microtome and stored at −20 °C for immunohistochemical analysis. Brain sections were washed three times with PBS, and then incubated in a blocking solution (0.3% Trition X-100 and 10% normal donkey serum in TBS) for 1 h. After that brain sections were incubated overnight at 4 °C with primary antibodies: rabbit anti-GFP (1:1000, Invitrogen, Carlsbad, CA, USA), followed by incubation with donkey anti-rabbit Alexa 488 (1:1000, Invitrogen) for 1 h at room temperature.

For evaluation of cell proliferation, mice were i.p. injected with 50 mg kg^−1^ 5-bromo-2-deoxyuridine (BrdU, Sigma Aldrich) in 0.9% NaCl at 0800 hours and perfused 2 h later. BrdU-positive cells represented the newborn cells. For estimating the number of newly generated neurons, mice were given four injections of BrdU every 24 h at 0800 hours and killed 4 weeks after the last injection. BrdU^+^/NeuN^+^ double-labeled cells were the survived newborn neurons. For BrdU staining, brain sections were pretreated with 2n HCl for 30 min and then washed in 0.1 m borate solution (pH 8.5) for 10 min. After incubation in a blocking solution, brain sections were incubated with primary antibodies: sheep anti-BrdU (1:1000, Abcam, Cambridge, MA, USA) and mouse anti-NeuN (1:1000, Millipore, Billerica, MA, USA), followed by donkey anti-sheep Alexa 594 (1:1000, Invitrogen) and donkey anti-mouse Alexa 488 (1:1000, Life Technologies, Carlsbad, CA, USA) for 1 h.

### Quantification and imaging

Images were acquired using a confocal fluorescence LSM-780 microscope (Carl Zeiss, Jena, Germany) fitted with standard filter sets and a standard (1 Airy disk) pinhole (Microstructural platform of Shandong University). BrdU^+^ and BrdU^+^NeuN^+^ cells were counted using the MetaMorph software package (Molecular Devices, Sunnyvale, CA, USA) with an unbiased stereological protocol as previously described.^33^ The observer was blind to the experimental design to prevent any inadvertent bias in cell counting. Every sixth section was counted for positive cells in the ventral dental gyrus (DG), which ensured that the same cell was not counted repeatedly.

### Quantitative real-time PCR

Mice were killed immediately after CRS. Brains were removed after decapitation and different brain regions were acquired using a mouse brain slicer. Total RNA was extracted using TRNzol-A^+^ RNA isolation reagent (Tiangen, Beijing, China) according to the manufacturer's instructions. Purified total RNA (500 ng) was then reverse transcribed to cDNA using the RevertAid First Strand cDNA Synthesis Kit (Fermentas, Burlington, ON, Canada). Quantitative Real-time PCR was performed in a Cycler (Bio-Rad, Hercules, CA, USA) using SYBR Green (Roche Diagnostics, Mannheim, Germany). Primer sequences used in the paper were listed in Supplementary Table S1. Each sample was analysed in duplicate and the relative levels of mRNA were normalized for each well of the β-actin mRNA levels using the 2^−ΔΔCT^ method.

### Statistical analysis

For comparison of two groups, data were analyzed by Student's *t-*test, followed by Bonferroni's correction. One-way analysis of variance (ANOVA) was performed for three or more groups, followed by the LSD *post hoc* test. When more than one factor was examined simultaneously, data were analyzed by two-way ANOVA, followed by the LSD *post hoc test*. For body weight, data were analyzed by repeated measures two-way ANOVA. A Dunnett's T3 test was used to compare the differences between groups when equal variances were not assumed. *P*-values<0.05 were considered statistically significant and results were represented as means±s.e.m. Data analyses were performed using SPSS statistical program, version 19.0 (IBM, Armonk, NY, USA).

## Results

### Selective reduction of Wnt2 and Wnt3 in the VH after CRS

CRS, a well-established animal model used to induce depression- and anxiety-like behaviors, was applied in our experiments.^19, 34, 35, 36, 37^ Mice were subjected to restraint treatment for 2 h per day for 14 days. Mice in the CRS group showed more anhedonic (that is, decreased sucrose consumption in SPT, *t*_14_=6.551, *P*<0.001, two-tailed *t*-test) and despair behaviors (that is, increased immobility in FST, (*t*_14_=−4.378, *P*=0.001, two-tailed *t*-test) and TST, (*t*_14_=−4.224, *P*=0.001, two-tailed *t*-test)) compared with the No-CRS group (Supplementary Figure S1A–C). In the open field test (OFT), there was no difference between the CRS group and the No-CRS group in the total distance traveled, indicating that locomotor activity was unaffected (Supplementary Figure S1D; *t*_14_=0.381, *P*=0.709, two-tailed *t*-test). Compared with the No-CRS group, mice in the CRS group spent less time in the center compartment (Supplementary Figure S1E; *t*_14_=2.544, *P*=0.034, two-tailed *t*-test). In elevated plus maze (EPM), the CRS group of mice also displayed a significant decrease in the percentage of time spent in the open arms (Supplementary Figure S1F; *t*_14_=5.987, *P*<0.001, two-tailed *t*-test) and in the percentage of entries into the open arms (Supplementary Figure S1G; *t*_14_=5.910, *P*<0.001, two-tailed *t*-test), which suggested that mice in the CRS group displayed anxiety-like behaviors. We also detected the effects of daily restraint stress on body weight. Prior to CRS exposure, the body weights of mice in the No-CRS and CRS groups were similar (Supplementary Figure S1H; *t*_14_=0.553, *P*=0.589). A two-way repeated ANOVA revealed that CRS had a significant effect on body weight, and that the stress × day interaction was significant (two-way repeated ANOVA: interaction: *F*_13,182_=46.298, *P*<0.001; stress: *F*_1,182_=7.808, *P*=0.027; day: *F*_13,182_=16.662, *P*<0.001). An LSD *post hoc test* revealed that the CRS group showed a significant decrease in body weight compared with the No-CRS group starting from the sixth day and lasting throughout the remainder of stress exposure days (*P*<0.05). These results validate the use of CRS as an animal depression and anxiety model in our experiments.

**Figure 1:**
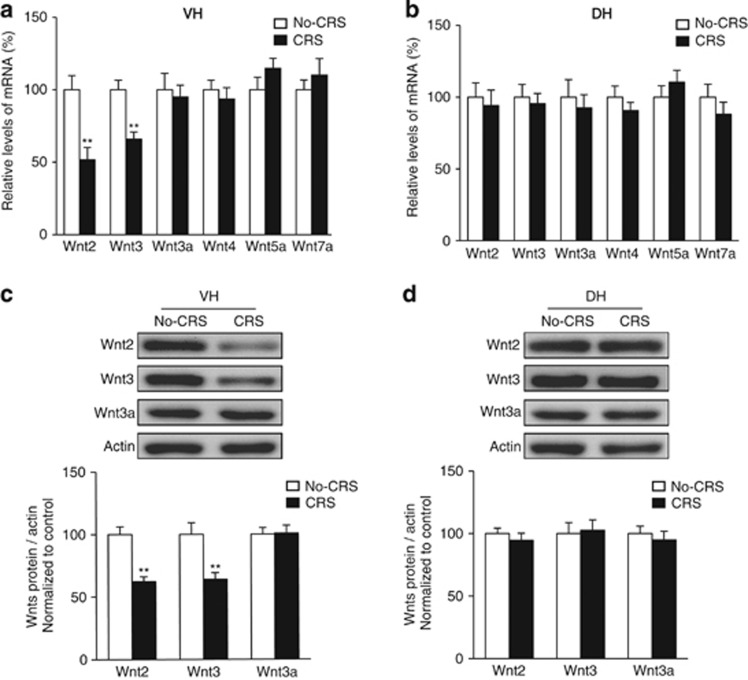
Selective reduction of Wnt2 and Wnt3 in the VH after CRS. (**a**) Changes in Wnt (2, 3, 3a, 4, 5a and 7a) mRNA levels in the VH after CRS. *n*=7 per group; **P<0.01 versus the No-CRS group. (**b**) Changes in Wnt (2, 3, 3a, 4, 5a and 7a) mRNA levels in the DH after CRS. *n*=7 per group. (**c**, **d**) Changes in Wnt (2, 3 and 3a) protein levels in the VH (**c**) and DH (**d**) after exposure to CRS, as detected by western blot. *n*=9 per group; **P<0.01 versus the No-CRS group. All values are denoted as the mean±s.e.m. CRS, chronic restraint stress; DH, dorsal hippocampus; VH, ventral hippocampus.

We initially examined whether the expression levels of various Wnts were altered after CRS. We focused on Wnt2, Wnt3, Wnt3a, Wnt4, Wnt5a and Wnt7a, which have been reported to be highly expressed in the hippocampus.^9, 38, 39, 40, 41^ Previous studies have shown that the DH appears to be more involved in learning and memory functions, whereas the VH seems to participate in emotional modulation.^42, 43^ Thus, we divided the hippocampus into the VH and DH according to the previous references,^27, 28^ and proved the veracity of these regions by differential expression of the Dcn and Lct further (Supplementary Figure S2). Real-time PCR results showed that among the six different Wnts, only Wnt2 and Wnt3 mRNA levels were significantly reduced in the VH (Figure 1a; Wnt2, *t*_12_=3.945, *P*=0.002; Wnt3, *t*_12_=4.342, *P*=0.001, two-tailed *t*-test). There were no significant changes in any of the six Wnts examined in the DH after CRS (Figure 1b). Certainly, there is another explanation for this result that a single restraint operation is powerful enough to induce the decrease of Wnt2/3 in the VH. To exclude the possibility, mice were subjected to a single restraint stress for 2 h and the mRNA levels of Wnt2/3 in the VH were detected immediately. The results revealed that there were no significant changes of Wnt2/3 mRNA (Supplementary Figure S3; Wnt2, *t*_18_=0.019, *P*=0.985; Wnt3, *t*_18_=−0.468, *P*=0.645, two-tailed *t*-test), suggesting that the decreased Wnt2/3 gene expression was specific to CRS.

We next determined whether the decrease observed in Wnt2 and Wnt3 mRNA levels would lead to reduction in protein levels after CRS. Western blots analysis showed that the protein levels of Wnt2 and Wnt3 were significantly decreased after CRS in the VH but not in the DH (Figure 1c; Wnt2, *t*_16_=5.175, *P*<0.001; Wnt3, *t*_16_=3.334, *P*=0.004, two-tailed *t*-test; Figure 1d; Wnt2, *t*_16_=0.766, *P*=0.455; Wnt3, *t*_16_=−0.213, *P*=0.834, two-tailed *t*-test). As an alternative control, we did not detect a significant alteration in the Wnt3a protein level in either the VH or the DH (Figures 1c and d).

The selective reduction of Wnt2 and Wnt3 expression in the VH following CRS suggested that Wnt2 and Wnt3 may play roles in CRS-induced depression-like behaviors.

### Knockdown of Wnt2 or Wnt3 in the No-CRS group could mimic depression-like behaviors

Our results showed that CRS could selectively decrease the expression of Wnt2 and Wnt3, so we next investigated whether endogenous Wnt2 and Wnt3 are functionally related to depression-like behaviors. We generated lentiviruses expressing siRNA sequences against Wnt2 or Wnt3 (Lenti-siWnt2 and Lenti-siWnt3) and a control lentivirus with a scrambled sequence (Lenti-siSCR). To exclude the potential off-target effect of siWnt2 and siWnt3, an overexpressed Wnt2 or Wnt3 HEK293 cells system was introduced. Respective siRNAs were transfected into the overexpressed HEK293 cells, 48 h later the Wnt2 and Wnt3 expression levels were detected by western blot. We found that the expression of Wnt2 but not Wnt3 was attenuated by 60% after transfection of Lenti-siWnt2. Similarly, Wnt3 expression was decreased after infection of Lenti-siWnt3, while Wnt2 was not affected (Supplementary Figures S5A and B: Supplementary Figure S5A; *F*_2,15_=22.287, *P*<0.001, one-way ANOVA; *post hoc* test, Lenti-siWnt2+Wnt2, *P*<0.001; Lenti-siWnt3+Wnt2, *P*=0.359; Supplementary Figure S5B; *F*_2,15_=16.379, *P*<0.001, one-way ANOVA; *post hoc* test, Lenti-siWnt2+Wnt3, *P*=0.564; Lenti-siWnt3+Wnt3, *P*<0.001). Then, mice were stereotactically injected with Lenti-siWnt2, Lenti-siWnt3 and Lenti-siSCR virus into the VH. The GFP gene was co-expressed with siRNA on the lentivirus vector. Therefore, the location of the microinjected lentivirus was showed by green fluorescence on the slices, while the effect of the virus was evaluated by the levels of Wnt2/3 protein with western blot. We carried these experiments every week after microinjection and found that there were a maximum GFP expression (Supplementary Figure S5C) and the lowest levels of Wnt2/3 at 5 weeks post injection (Supplementary Figure S5D; Wnt2, *t*_11_=6.677, *P*<0.001; Wnt3, *t*_11_=6.349, *P*<0.001, two-tailed *t*-test), indicating that the effect of virus was optimal at this time point. Therefore, in the following studies, behavioral tests were performed 5 weeks after virus injection.

**Figure 2:**
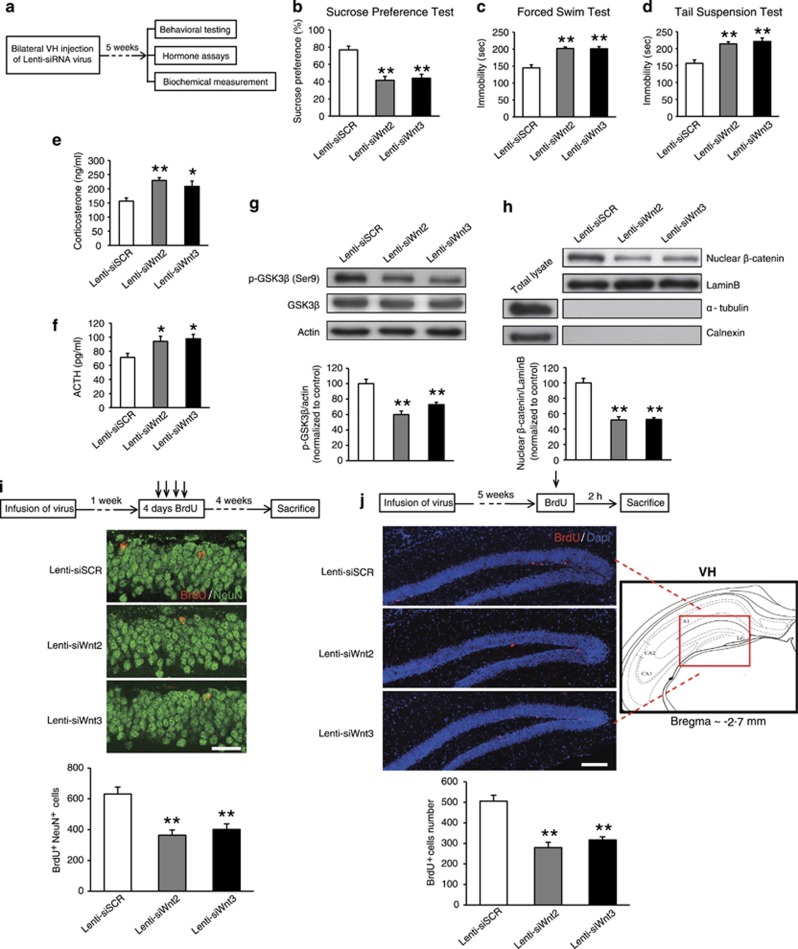
Knockdown of Wnt2 or Wnt3 in the VH could mimic depression-like behaviors. (**a**) Schematic of the experimental schedule used to investigate the effect of siWnt2 and siWnt3 virus injections into the VH on depression behaviors and hormone levels. The effects of Lenti-siWnt2 or Lenti-siWnt3 on depression-like behaviors in mice were examined, including (**b**) sucrose consumption in sucrose preference test (SPT), (**c**) immobility time in forced swim test (FST) and (**d**) immobility time in tail suspension test (TST). *n*=10 per group; *P<0.05, **P<0.01 versus the Lenti-siSCR group. (**e**,**f**) Lenti-siWnt2 or Lenti-siWnt3 resulted in higher serum corticosterone (**e**) and ACTH (**f**) levels compared with Lenti-siSCR group. Lenti-siSCR group: *n*=5; Lenti-siWnt2 group: *n*=6; Lenti-siWnt3 group: *n*=6. *P<0.05, **P<0.01 versus the Lenti-siSCR group. (**g**) Representative blots and densitometric analysis of p-GSK3β (Ser9) in the cytoplasmic fractions of VH after knockdown of Wnt2 or Wnt3. *n*=6 per group; **P<0.01 versus the Lenti-siSCR group. (**h**) Representative blots and densitometric analysis of β-catenin in the nuclear fractions of VH after knockdown of Wnt2 or Wnt3. *n*=6 per group; **P<0.01 versus the Lenti-siSCR group. (**i**) Knockdown of either Wnt2 or Wnt3 decreased the number of newborn neuron. Representative images showed BrdU^+^NeuN^+^ cells in the DG (scale bar, 20 μm) and quantification of BrdU^+^NeuN^+^ cells. *n*=5 per group; **P<0.01 versus the Lenti-siSCR group. (**j**) Knockdown of either Wnt2 or Wnt3 impaired cell proliferation. Left, representative images showed BrdU^+^ cells in the DG (scale bar, 200 μm) and quantification of BrdU^+^ cells. Right, a schematic diagram from ref. 26, which represents the VH. *n*=6 per group; **P<0.01 versus the Lenti-siSCR group. All values are denoted as the mean±s.e.m. ACTH, adrenocorticotropin hormone; DG, dental gyrus; VH, ventral hippocampus.

We next investigated whether decreasing Wnt2 or Wnt3 could mimic depression-like behaviors (Figure 2a). Mice in the Lenti-siWnt2 and Lenti-siWnt3 groups showed decreased sucrose consumption compared with mice in the Lenti-siSCR group (Figure 2b; *F*_2,27_=19.394, *P*<0.001, one-way ANOVA; *post hoc* test, Lenti-siWnt2, *P*<0.001; Lenti-siWnt3, *P*<0.001). There was a significant increase in immobility time in both FST (Figure 2c; *F*_2,27_=21.964, *P*<0.001, one-way ANOVA; *post hoc* test, Lenti-siWnt2, *P*<0.001; Lenti-siWnt3, *P*<0.001) and TST (Figure 2d; *F*_2,27_=15.686, *P*<0.001, one-way ANOVA; *post hoc* test, Lenti-siWnt2, *P*<0.001; Lenti-siWnt3, *P*<0.001) in the Lenti-siWnt2 and Lenti-siWnt3 groups compared with the Lenti-siSCR group. In the OFT, the Lenti-siWnt2 and Lenti-siWnt3 groups exhibited no significant difference in locomotor activity or in the percentage of time spent in the central areas compared with the Lenti-siSCR group (Supplementary Figure S5E; *F*_2,27_=0.476, *P*=0.627, one-way ANOVA; *post hoc* test, Lenti-siWnt2, *P*=0.383; Lenti-siWnt3, *P*=0.928; Supplementary Figure S5F; *F*_2,27_=0.430, *P*=0.655, one-way ANOVA; *post hoc* test, Lenti-siWnt2, *P*=0.362; Lenti-siWnt3, *P*=0.625). In EPM, neither the percentage of time spent in the open arms (Supplementary Figure S5G; *F*_2,27_=0.480, *P*=0.624, one-way ANOVA; *post hoc* test, Lenti-siWnt2, *P*=0.445; Lenti-siWnt3, *P*=0.895) nor the percentage of entries into the open arms (Supplementary Figure S5H; *F*_2,27_=1.153, *P*=0.331, one-way ANOVA; *post hoc* test, Lenti-siWnt2, *P*=0.826; Lenti-siWnt3, *P*=0.244) was significantly different among Lenti-siWnt2, Lenti-siWnt3 and Lenti-siSCR groups. Together, our results suggested that knockdown of Wnt2 or Wnt3 expression in the VH could mimic CRS-induced depression-like, but not anxiety-like behaviors. Previous studies have provided extensive evidence for the hypothalamic–pituitary–adrenal axis dysregulation in depression.^44, 45^ We next detected changes in serum corticosterone and adrenocorticotropin hormone (ACTH) levels following knockdown of Wnt2 or Wnt3. As shown in Figures 2e and f, knockdown of either Wnt2 or Wnt3 resulted in a significant increase in corticosterone and ACTH levels compared with Lenti-siSCR group (corticosterone; *F*_2,14_=6.773, *P*=0.009, one-way ANOVA; *post hoc* test, Lenti-siWnt2, *P*=0.003; Lenti-siWnt3, *P*=0.022; ACTH; *F*_2,14_=4.627, *P*=0.029, one-way ANOVA; *post hoc* test, Lenti-siWnt2, *P*=0.029; Lenti-siWnt3, *P*=0.013).

**Figure 3:**
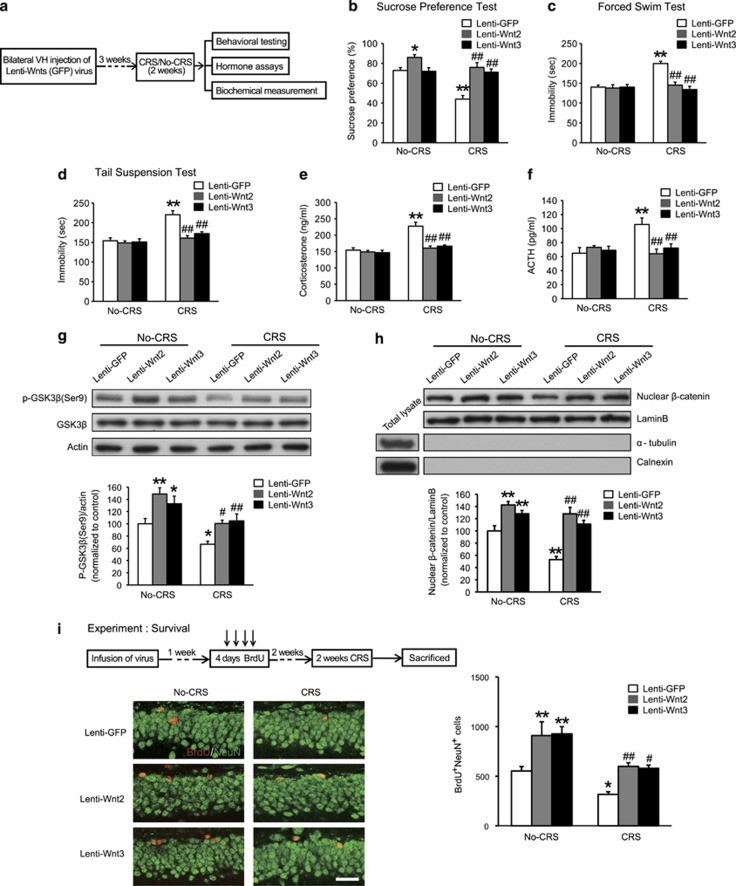
Wnt2 and Wnt3 buffer CRS-induced depression-like behaviors. (**a**) Schematic of the experimental schedule used to investigate the effect of Wnt2 or Wnt3 overexpression in the VH on depression-like behaviors and hormone levels under basal or stress condition. The effect of Lenti-Wnt2 and Lenti-Wnt3 viruses on (**b**) sucrose consumption in SPT, (**c**) immobility time in FST, and (**d**) immobility time in TST under basal or stress condition. *n*=10 per group; *P<0.05, **P<0.01 versus the Lenti-GFP group; ^##^*P*<0.01 versus the CRS-Lenti-GFP. (**e, f**) The effect of overexpression of Wnt2 or Wnt3 on serum corticosterone (**e**) and ACTH (**f**) levels under basal or stress condition. *n*=5, 6, 7, 6, 8, 6, respectively; **P<0.01 versus the Lenti-GFP group; ^##^*P*<0.01 versus the CRS-Lenti-GFP group. (**g**) Representative blots and densitometric analysis of p-GSK3β (Ser9) in the cytoplasmic fractions of VH after overexpression of Wnt2 or Wnt3. under basal or stress condition. *n*=7 per group; *P<0.05, **P<0.01 versus the Lenti-GFP; ^#^*P*<0.05, ^##^*P*<0.01 versus the CRS-Lenti-GFP group. (**h**) Representative blots and densitometric analysis of β-catenin in the nuclear fractions of VH after overexpression of Wnt2 or Wnt3 under basal or stress condition. *n*=7 per group; *P<0.05, **P<0.01 versus the Lenti-GFP; ^#^*P*<0.05, ^##^*P*<0.01 versus the CRS-Lenti-GFP group. (**i**) Overexpression of Wnt2 or Wnt3 rescued newborn neuron survival under basal or stress condition. Left, representative images showed BrdU^+^NeuN^+^ cells in the DG (scale bar, 20 μm). Right, quantification of BrdU^+^NeuN^+^ cells. *n*=5 per group; *P<0.05, **P<0.01 versus the Lenti-GFP group; ^#^*P*<0.05, ^##^*P*<0.01 versus the CRS-Lenti-GFP group. All values are denoted as the mean±s.e.m. ACTH, adrenocorticotropin hormone; CRS, chronic restraint stress; DG, dental gyrus; FST, forced swim test; GFP, green-fluorescent protein; SPT, sucrose preference test; TST, tail suspension test; VH, ventral hippocampus.

We next investigated the mechanisms underlying Wnt2- or Wnt3-mediated depression-like behaviors. Previous studies have reported that Wnt/β-catenin signaling regulates neurogenesis and is associated with depression disorders.^9, 11^ We therefore first examined whether the Wnt/β-catenin signaling pathway was altered at 5 weeks after the injection of the Lenti-siWnt2, Lenti-siWnt3 or Lenti-siSCR virus. Western blot analysis showed that the total GSK3β levels were not significantly changed. However, Lenti-siWnt2 or Lenti-siWnt3 injection significantly decreased the level of p-GSK3β (Ser9) and nuclear β-catenin compared with Lenti-siSCR injection (Figure 2g; p-GSK3β: *F*_2,15_=19.717, *P*<0.001, one-way ANOVA; *post hoc* test, Lenti-siWnt2, *P*<0.001; Lenti-siWnt3, *P*=0.001; Figure 2h; nuclear β-catenin: *F*_2,15_=39.312, *P*<0.001, one-way ANOVA; *post hoc* test, Lenti-siWnt2, *P*<0.001; Lenti-siWnt3, *P*<0.001), which suggested that knockdown of Wnt2 or Wnt3 led to impaired Wnt/β-catenin signaling. The nuclear fraction we purified had no expression of α-tubulin (the cytosolic marker) and calnexin (the membrane marker), which excluded the possibility of the contamination by cytoplasmic and membrane proteins (Figure 2h and Figure 3h).

We further investigated whether knockdown of Wnt2 or Wnt3 would lead to impaired neurogenesis. First, the number of surviving newborn neurons was analysed by cells that were double-labeled for BrdU and NeuN 4 weeks after the last BrdU injection. The results showed that CRS could significantly decrease survived newborn neurons in the VH but not DH (Supplementary Figure S6; DH: *t*_7_=0.351, *P*=0.736, two-tailed *t*-test; VH: *t*_7_=5.637, *P*=0.006, two-tailed *t*-tes). Therefore, we focused on the alteration of adult neurogenesis in the VH in the following experiments. Similarly, knockdown of Wnt2 or Wnt3 could lead to a significant reduction in BrdU^+^NeuN^+^ cells, compared with Lenti-siSCR group (Figure 2i; *F*_2,12_=14.071, *P*=0.001, one-way ANOVA; *post hoc* test, Lenti-siWnt2, *P*<0.001; Lenti-siWnt3, *P*=0.001), suggesting that knockdown of Wnt2 or Wnt3 decreases the number of surviving hippocampal newborn neurons. Then, we evaluated whether the decreased number of surviving hippocampal newborn neurons was related with the decrease of cell proliferation. The result showed that there was a significant decrease in BrdU^+^-labeled cells 2 h after BrdU injection in the Lenti-siWnt2 or Lenti-siWnt3 group compared with Lenti-siSCR group (Figure 2j; *F*_2,15_=26.248, *P*<0.001, one-way ANOVA; *post hoc* test, Lenti-siWnt2, *P*<0.001; Lenti-siWnt3, *P*<0.001), which suggested that knockdown of Wnt2 or Wnt3 impaired cell proliferation in the adult VH.

Together, our results demonstrated that knockdown of Wnt2 or Wnt3 in the VH could lead to depression-like behaviors, impaired Wnt/β-catenin signaling and decreased neurogenesis, which suggested that the induced depression-like behaviors may be the results of impaired Wnt/β-catenin signaling or decreased neurogenesis in the VH.

### Wnt2 and Wnt3 could buffer CRS-induced depression-like behaviors

We next tested whether overexpressing Wnt2 or Wnt3 in the VH is sufficient for alleviating CRS-induced depression-like behaviors. We injected lentiviruses expressing Wnt2 or Wnt3 into the VH of adult mice (Figure 3a). First, we evaluated the effect of the Lenti-Wnt2 and Lenti-Wnt3 viruses on the expression of Wnt2 and Wnt3 (Supplementary Figure S7A). Western blot results showed that Wnt2 and Wnt3 levels were significantly increased 5 weeks after injection of Lenti-Wnt2 and Lenti-Wnt3, respectively, compared with injections of Lenti-GFP in the VH (Supplementary Figure S7A; Wnt2, *t*_9_=−2.731, *P*=0.023; Wnt3, *t*_10_=−4.029, *P*=0.002, two-tailed *t*-test).

Under basal condition, behavioral tests were performed 5 weeks after virus injection (Figure 3a). We found that sucrose consumption was significantly increased in the Lenti-Wnt2 group (Figure 3b; *P*=0.012). However, there was no significant difference in the immobility time in FST and TST among the Lenti-Wnt2, Lenti-Wnt3 and Lenti-GFP groups of No-CRS mice (Figures 3c and d). As expected, under stress condition, the CRS-Lenti-GFP group showed depression-like behaviors compared with the Lenti-GFP group (Figures 3b–d; *P<*0.001). Interestingly, we found that sucrose consumption was significantly increased in the CRS-Lenti-Wnt2 and CRS-Lenti-Wnt3 groups compared with the CRS-Lenti-GFP group (Figure 3b; *P*<0.001). Analysis by two-way ANOVA found a significant stress × virus interaction effect and a significant effect of stress and virus (two-way ANOVA: interaction: *F*_2,54_=7.946, *P*=0.001; stress: *F*_1,54_=20.384, *P*<0.001; virus: *F*_2,54_=20.010, *P*<0.001). Mice in the CRS-Lenti-Wnt2 or CRS-Lenti-Wnt3 group displayed significantly decreased immobility time in FST and TST compared with the CRS-Lenti-GFP group (Figures 3c and d; *P<*0.001). Two-way ANOVA revealed a significant effect of stress and virus in FST and TST, where the effect of virus was dependent on CRS (two-way ANOVA: FST: Interaction: *F*_2,54_=12.627, *P*<0.001; stress: *F*_1,54_=13.166, *P*=0.001; virus: *F*_2,54_=13.362, *P*<0.001; TST: Interaction: *F*_2,54_=5.599, *P*=0.006; stress: *F*_1,54_=8.588, *P*=0.005; virus: *F*_2,54_=8.638, *P*=0.001). Our results suggested that the effects of overexpressing Wnt2 and Wnt3 more specifically resulted in a reversal of stress-induced depression-like behaviors.

We also investigated the role of Wnt2 and Wnt3 in anxiety-like behaviors. In OFT, there was no significant difference in total distance traveled among the Lenti-Wnt2, Lenti-Wnt3 and Lenti-GFP groups under basal or stress condition, indicating that locomotor activity was unaffected (Supplementary Figure S7B). No significant stress × virus interaction effect or virus effect was observed between the groups for the percentage of time spent in the center, but a significant effect for stress treatment was noted (Supplementary Figure S7C; two-way ANOVA: interaction: *F*_2,54_=0.488, *P*=0.617; stress: *F*_1,54_=69.602, *P*<0.001; virus: *F*_2,54_=0.134, *P*=0.875). In EPM, no significant stress × virus interaction effect or virus effect was observed between groups for percentage of time spent in or entries into open arms; however, a significant effect of stress was found (Supplementary Figure S7D; two-way ANOVA: interaction: *F*_2,54_=0.112, *P*=0.894; stress: *F*_1,54_=64.419, *P*<0.001; virus: *F*_2,54_=0.513, *P*=0.602; Supplementary Figure S7E; two-way ANOVA: interaction: *F*_2,54_=0.415, *P*=0.662; stress: *F*_1,54_=115.221, *P*<0.001; virus: *F*_2,54_=0.388, *P*=0.680). Together, these data suggested that Wnt2 and Wnt3 had no effect on locomotor activity or anxiety-like behaviors.

In addition, we detected serum corticosterone and ACTH levels (Figure 3a). We found that overexpression of Wnt2 or Wnt3 had no effect on serum corticosterone or ACTH levels under basal condition, whereas overexpression could reverse the CRS-induced increase in serum levels of corticosterone and ACTH (Figure 3e; CRS-Lenti-Wnt2, *P*<0.001; CRS-Lenti-Wnt3, *P*<0.001; Figure 3f; CRS-Lenti-Wnt2, *P*<0.001; CRS-Lenti-Wnt3, *P*=0.001). Analysis by two-way ANOVA indicated a significant stress × virus interaction effect and a significant effect of stress and virus (corticosterone; two-way ANOVA: interaction: *F*_2,32_=9.532, *P*=0.001; stress: *F*_1,32_=33.674, *P*<0.001; virus: *F*_2,32_=14.243, *P*<0.001; ACTH; two-way ANOVA: interaction: *F*_2,32_=7.176, *P*=0.003; stress: *F*_1,32_=4.589, *P*=0.04; virus: *F*_2,32_=3.490, *P*=0.04).

Our results demonstrated that overexpressing Wnt2 or Wnt3 was sufficient for alleviating CRS-induced depression-like, but not anxiety-like behaviors.

### Wnt2 and Wnt3 rescue CRS-induced Wnt/β-catenin signaling impairment and deficits in neurogenesis

Previous studies reported that chronic stress leads to Wnt/β-catenin signaling impairment and deficits in neurogenesis.^22, 46^ Our results, described above, showed that overexpressing Wnt2 and Wnt3 rescued CRS-induced depression-like behaviors. Therefore, we next examined whether overexpressing Wnt2 or Wnt3 could rescue the CRS-induced Wnt/β-catenin signaling impairment and deficits in neurogenesis. We found that the levels of p-GSK3β (Ser9) and nuclear β-catenin were significantly increased in the Lenti-Wnt2 or Lenti-Wnt3 group compared with the Lenti-GFP group under basal condition, which suggested that overexpressing Wnt2 or Wnt3 enhanced Wnt/β-catenin signaling (Figure 3g; p-GSK3β: Lenti-Wnt2, *P*=0.001; Lenti-Wnt3, *P*=0.016; Figure 3h; β-catenin: Lenti-Wnt2, *P*<0.001; Lenti-Wnt3, *P*=0.009). Under stress condition, overexpression of Wnt2 or Wnt3 could rescue the CRS-induced decreases in p-GSK3β (Ser9) (CRS-Lenti-Wnt2, *P*=0.013; CRS-Lenti-Wnt3, *P*=0.005) and nuclear β-catenin (CRS-Lenti-Wnt2, *P*<0.001; CRS-Lenti-Wnt3, *P*<0.001), suggesting that Wnt2 and Wnt3 rescued the CRS-induced Wnt/β-catenin signaling impairment.

Previous studies reported that overexpression of Wnt3 was sufficient for increasing neurogenesis *in vitro* and *in vivo*.^9^ However, whether Wnt2 is involved in adult neurogenesis remains unclear. Our results showed that the number of BrdU^+^NeuN^+^ cells was significantly increased in the Lenti-Wnt2 or Lenti-Wnt3 group compared with the Lenti-GFP group under basal condition (Figure 3i; Lenti-Wnt2, *P*=0.002; Lenti-Wnt3, *P*=0.001), which suggests that Wnt2 and Wnt3 are involved in adult neurogenesis. Under stress condition, overexpression of Wnt2 or Wnt3 blocked the CRS-induced decrease in BrdU^+^NeuN^+^ cells compared with the CRS-Lenti-GFP group (Figure 3i; CRS-Lenti-Wnt2, *P*=0.010; CRS-Lenti-Wnt3, *P*=0.015), which suggested that overexpression of Wnt2 or Wnt3 rescued CRS-induced hippocampal neurogenesis deficits. The above results indicated that Wnt/β-catenin signaling and hippocampal neurogenesis, coupled with Wnt2 and Wnt3, may mediate CRS-induced depression-like behaviors.

### Wnt2 and Wnt3 positively regulate each other via CREB

**Figure 4:**
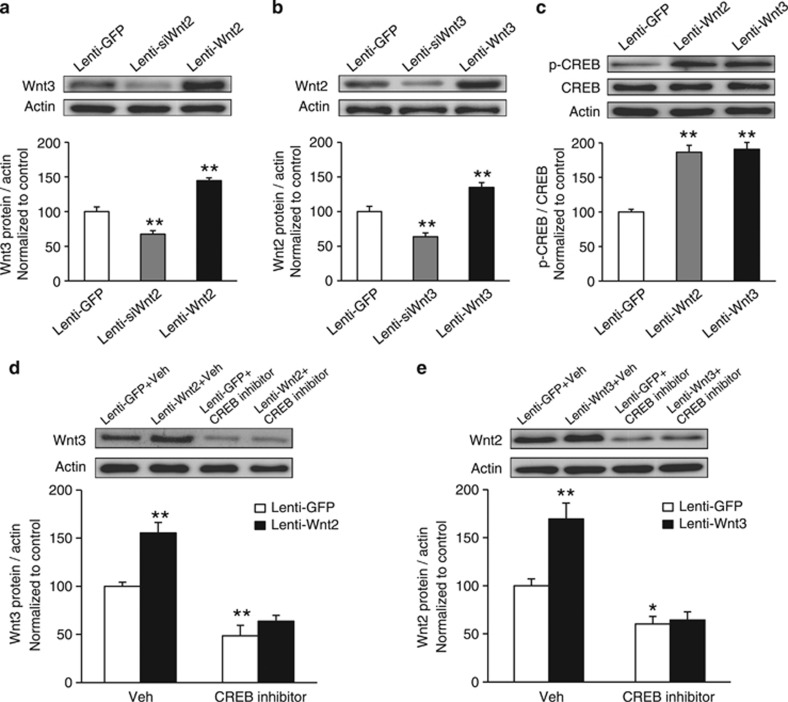
Wnt2 and Wnt3 positively regulate each other via CREB. (**a**) Levels of Wnt3 in the VH of mice injected with Lenti-GFP, Lenti-siWnt2 or Lenti-Wnt2, analyzed by western blot. *n*=6 per group; **P<0.01 versus the Lenti-GFP group. (**b**) Levels of Wnt2 in the VH of mice injected with Lenti-GFP, Lenti-siWnt3 or Lenti-Wnt3, analyzed by western blot. *n*=6 per group; **P<0.01 versus the Lenti-GFP group. (**c**) Representative blots and densitometric analysis showing p-CREB levels in cultured neurons that overexpressed Wnt2 or Wnt3. *n*=5 per group; **P<0.01 versus the Lenti-GFP group. (**d**) Representative blots and densitometric analysis showing Wnt3 levels in cultured neurons that overexpressed Wnt2 after administration of a CREB inhibitor. *n*=4, 5, 4, 4, respectively; **P<0.01 versus the Lenti-GFP+Vehicle (Veh) group. (**e**) Representative blots and densitometric analysis showing Wnt2 levels in cultured neurons that overexpressed Wnt3 after administration of a CREB inhibitor. *n*=6, 6, 5, 5, respectively; *P<0.05, **P<0.01 versus the Lenti-GFP+Veh group. All values are denoted as the mean±s.e.m. CREB, cAMP response element-binding protein; GFP, green-fluorescent protein; VH, ventral hippocampus.

Our above experiments showed that CRS induces a synchronous reduction in Wnt2 and Wnt3, and that Wnt2 and Wnt3 participate in depression-like behaviors. We next sought to determine the relationship between Wnt2 and Wnt3. In our experiments, we found that knockdown of Wnt2 decreased, whereas overexpression of Wnt2 increased Wnt3 levels compared with the Lenti-GFP group *in vivo* and vice versa (Figure 4a; *F*_2,15_=51.806, *P*<0.001, one-way ANOVA; *post hoc* test, Lenti-siWnt2, *P*=0.001; Lenti-Wnt2, *P*<0.001; Figure 4b; *F*_2,15_=27.506, *P*<0.001, one-way ANOVA; *post hoc* test, Lenti-siWnt3, *P*=0.002; Lenti-Wnt3, *P*=0.002). Moreover, the potential off-target effect of siWnt2 and siWnt3 could be excluded (Supplementary Figures S5A and B). These results suggested that the expression levels of Wnt2 and Wnt3 are associated with each other.

Previous studies have reported that transcription of both Wnt2 and Wnt3 is CREB dependent.^47, 48^ Cultured neurons (7 DIV) were infected with Lenti-Wnt2 or Lenti-Wnt3 virus for 3 days, and we observed that although the total CREB levels exhibited no significant alteration, the levels of p-CREB were significantly increased after Lenti-Wnt2 or Lenti-Wnt3 virus infection, which suggested that both Wnt2 and Wnt3 activated CREB (Figure 4c; *F*_2,12_=36.713, *P*<0.001, one-way ANOVA; *post hoc* test, Lenti-Wnt2, *P*<0.001; Lenti-Wnt3, *P*<0.001). Furthermore, we found that administration of a CREB inhibitor (CBP–CREB interaction inhibitor) blocked the positive feedback effect between Wnt2 and Wnt3 (Figure 4d; *P=*0.002; Figure 4e; *P=*0.022). Two-way ANOVA analysis found a significant virus × inhibitor interaction effect and a significant effect of virus and inhibitor (two-way ANOVA: Figure 4d; Interaction: *F*_1,13_=4.922, *P*=0.04; virus: *F*_1,13_=15.044, *P*=0.002; inhibitor: *F*_1,13_=62.161, *P*<0.001; Figure 4e; Interaction: *F*_1,18_=8.475, *P*=0.009; virus: *F*_1,18_=10.781, *P*=0.004; inhibitor: *F*_1,18_=41.585, *P*<0.001). These results suggested that there is positive feedback between Wnt2 and Wnt3 that depends on the activation of CREB.

### Wnt2 and Wnt3 mediate the effect of antidepressant

**Figure 5:**
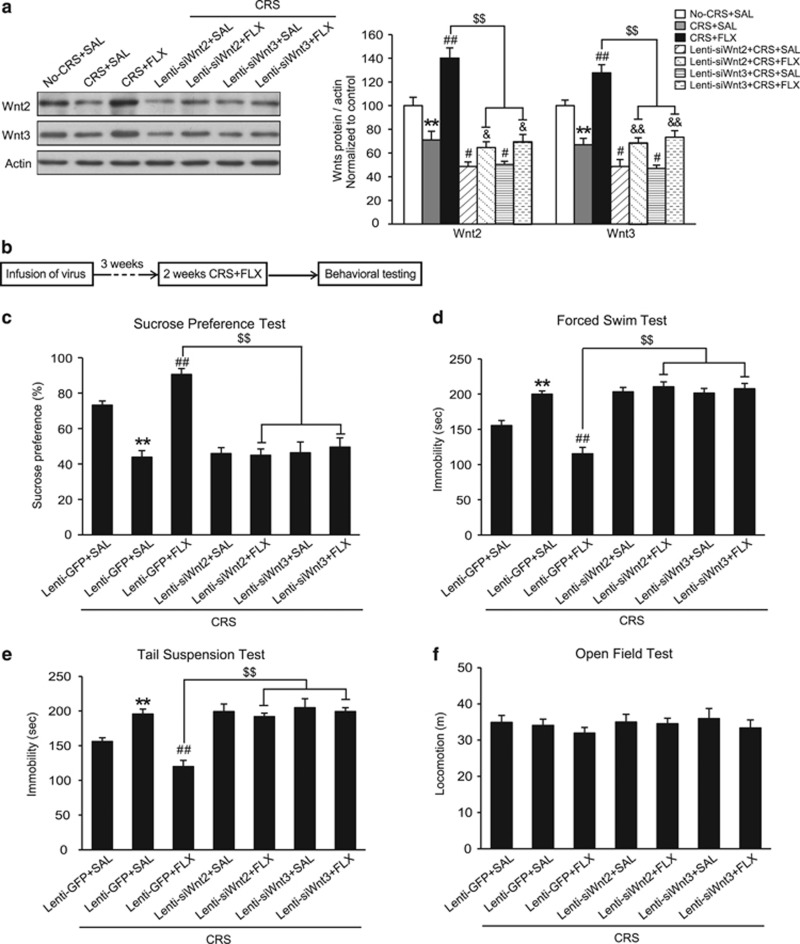
Wnt2 and Wnt3 mediate the effect of antidepressants. (**a**) Representative blots and densitometric analysis showing Wnt2 and Wnt3 protein levels in the VH of mice within the No-CRS+SAL, CRS+SAL, CRS+FLX, Lenti-siWnt2+CRS+SAL, Lenti-siWnt2+CRS+FLX, Lenti-siWnt3+CRS+SAL and Lenti-siWnt3+CRS+FLX treatment groups. *n*=7, 6, 7, 8, 8, 8, respectively; **P<0.01 versus the No-CRS+SAL group; ^#^*P*<0.05, ^##^*P*<0.01 versus the CRS+SAL group; ^$$^*P*<0.01 versus the CRS+FLX group; ^&^*P*<0.05, ^&&^*P*<0.01 versus the Lenti-siWnt2+CRS+SAL or Lenti-siWnt3+CRS+SAL group. (**b**) Schematic showing the experimental schedule used to investigate the effect of Wnt2 and Wnt3 on antidepressant responses to fluoxetine. (**c**) Sucrose consumption in SPT, (**d**) immobility time in FST and (**e**) immobility time in TST for mice in the Lenti-GFP+SAL, Lenti-GFP+CRS+SAL, Lenti-GFP+CRS+FLX, Lenti-siWnt2+CRS+SAL, Lenti-siWnt2+CRS+FLX, Lenti-siWnt3+CRS+SAL and Lenti-siWnt3+CRS+FLX groups. **P<0.01 versus the Lenti-GFP+SAL group; ^##^*P*<0.01 versus the CRS+Lenti-GFP+SAL group; ^$$^*P*<0.01 versus the CRS+Lenti-GFP+FLX group. (**f**) In the OFT, all groups showed no difference in locomotor activity. *n*=10, 10, 10, 9, 10, 7, 10, respectively. All values are denoted as the mean±s.e.m. CRS, chronic restraint stress; FLX, fluoxetine; FST, forced swim test; GFP, green-fluorescent protein; OFT, open field test; SAL, saline; SPT, sucrose preference test; VH, ventral hippocampus.

Previous studies have reported that Wnt2 expression and Wnt/β-catenin signaling are increased by different classes of antidepressant treatments and by electroconvulsive seizures under basal condition.^49, 50^ However, whether Wnt2 is necessary for the effect of antidepressants remains unclear. Previous studies have reported that chronic fluoxetine treatment reverses chronic stress-induced depression-like behaviors.^51, 52^ Here, we investigated whether Wnt2 or Wnt3 is necessary for the effects of fluoxetine treatment under stress condition. To examine whether Wnt2 or Wnt3 expression is altered in response to fluoxetine treatment under CRS condition, mice were randomly assigned into three groups: No-CRS+saline (No-CRS+SAL), CRS+saline (CRS+SAL) and CRS+fluoxetine (CRS+FLX). After 2 weeks of CRS and fluoxetine treatment, mice were killed, and the protein levels of Wnt2 and Wnt3 were detected. We found that two weeks of i.p. injections of fluoxetine significantly reversed the CRS-induced decrease in Wnt2 and Wnt3 levels compared with CRS+SAL group (Figure 5a; Wnt2: *F*_6,45_=29.602, *P*<0.001, one-way ANOVA; *post hoc* test, *P*=0.002; Wnt3: *F*_6,45_=30.120, *P*<0.001, one-way ANOVA; *post hoc* test, *P*<0.001), suggesting that Wnt2 and Wnt3 may be involved in the fluoxetine effect. We further examined the role of Wnt2 or Wnt3 in antidepressant effects by using lentivirus-mediated knockdown of Wnt2 or Wnt3 (Figure 5b). We found that the mice in the Lenti-siWnt2+SAL+CRS or Lenti-siWnt3+SAL+CRS group exhibited similar sucrose consumption in SPT (Figure 5c; *F*_6,59_=22.353, *P*<0.001, one-way ANOVA; *post hoc* test, Lenti-siWnt2+SAL+CRS, *P*=0.715, Lenti-siWnt3+SAL+CRS, *P*=0.689) and similar immobility times in FST (Figure 5d; *F*_6,59_=27.039, *P*<0.001, one-way ANOVA; *post hoc* test, Lenti-siWnt2+SAL+CRS, *P*=0.723, Lenti-siWnt3+SAL+CRS, *P*=0.879) and TST (Figure 5e; *F*_6,59_=16.472, *P*<0.001, one-way ANOVA; *post hoc* test, Lenti-siWnt2+SAL+CRS, *P*=0.756, Lenti-siWnt3+SAL+CRS, *P*=0.443) compared with the Lenti-GFP+SAL+CRS mice, which suggested that knockdown of Wnt2 or Wnt3 did not exacerbate CRS-induced depression-like behaviors. Treatment with fluoxetine counteracted the depression-like behaviors induced by CRS (Figures 5c–e; *P*<0.001). However, mice in the Lenti-siWnt2+FLX+CRS or Lenti-siWnt3+FLX+CRS group showed significantly decreased sucrose consumption in SPT and increased immobility times in FST and TST compared with the Lenti-GFP+FLX+CRS group (Figures 5c–e; Lenti-siWnt2+FLX+CRS, *P*<0.001, Lenti-siWnt3+FLX+CRS, *P*<0.001). Moreover, the Lenti-siWnt2+FLX+CRS or the Lenti-siWnt3+FLX+CRS group displayed no significant difference in sucrose consumption or immobility times in FST and TST compared with the Lenti-siWnt2+SAL+CRS group or the Lenti-siWnt3+SAL+CRS group. These findings suggested that knockdown of Wnt2 or Wnt3 could abolish the antidepressant effect of fluoxetine. We also found that combined stress and knockdown of Wnt2/3 could not cause a more severe phenotype (Figures 5c–e), which may be resulted from the presence of a basement effect in the experiments.

In addition, we found that there was a synchronous decrease of Wnt2 and Wnt3 in both the Lenti-siWnt2+CRS+SAL group and the Lenti-siWnt3+CRS+SAL group compared with the CRS+SAL group (Figure 5a; Wnt2: Lenti-siWnt2+CRS+SAL, *P*=0.013, Lenti-siWnt3+CRS+SAL, *P*=0.021; Wnt3: Lenti-siWnt2+CRS+SAL, *P*=0.019, Lenti-siWnt3+CRS+SAL, *P*=0.011). The fluoxetine-induced increase in Wnt2 and Wnt3 was also synchronously blocked in both the Lenti-siWnt2+CRS+FLX group and the Lenti-siWnt3+CRS+FLX group compared with the CRS+FLX group (Figure 5a; Wnt2: Lenti-siWnt2+CRS+FLX, *P*<0.001, Lenti-siWnt3+CRS+FLX, *P*<0.001; Wnt3: Lenti-siWnt2+CRS+FLX, *P*<0.001, Lenti-siWnt3+CRS+FLX, *P*<0.001). These results further confirmed that the expression levels of Wnt2 and Wnt3 are associated with each other.

## Discussion

In the present study, we showed that CRS exposure selectively decreased Wnt2 and Wnt3 expression in the VH but not in the DH, and that knocking down Wnt2 or Wnt3 could mimic depression-like behaviors. Moreover, overexpression of Wnt2 or Wnt3 in the VH was sufficient to buffer CRS-induced depression-like behaviors. Wnt2 and Wnt3 could activate CREB and we showed evidence for CREB-dependent positive feedback between Wnt2 and Wnt3. Finally, we determined that Wnt2 and Wnt3 in the VH could mediate the antidepressant effect of fluoxetine.

Previous studies have shown that Wnt/β-catenin signaling is involved in depression- but not anxiety-like behaviors by using β-catenin overexpressing transgenic mice or conditional β-catenin knockout mice.^16, 17^ Our data provided several new insights into the roles of Wnt2 and Wnt3 in CRS-induced depression-like behaviors. First, we found that CRS exposure could selectively induce decreased Wnt2 and Wnt3 expression in the VH but not in the DH. A previous study reported that Dkk-1, an inhibitor of the canonical Wnt pathway, is increased after exposure to stress.^22^ Wilkinson *et al.*^21^ also found that one member of the Wnt-DVL-GSK3β pathway, DVL, was downregulated by social defeat stress. Chronic unpredictable stress decreased the expression of Fzd6, one of the receptors involved in Wnt signaling.^20^ These studies suggest that Wnt signaling pathway is involved in stress-induced depression-like behaviors. However, the studies about the effects of wnt ligands on the pathogenesis, development and treatment of depression are not available. A microarray studies of the hippocampus after CRS have identified decreases in the Wnt2 transcript,^23^ and Okamato *et al.*^49^ have proved that Wnt2 is a common target of different classes of antidepressants, but there is no direct evidence to verify the functional correlation of Wnts and depression caused by stress. With the aid of positive and negative regulation of Wnt2/3, our study indicated a specific involvement of Wnt2 and Wnt3 in CRS for the first time. Moreover, we found that knockdown of Wnt2 or Wnt3 could mimic the depression- but not anxiety-like behaviors. Consistent with our results, knockdown of Fzd6 by injection of shRNA virus into the hippocampus resulted in depression-like behaviors in SPT.^20^

Increasing corticosterone levels could induce depression-like behaviors and decrease hippocampal neurogenesis.^44, 53, 54^ Our results showed that knockdown of Wnt2 or Wnt3 increased serum corticosterone and ACTH levels, which suggested that corticosterone and ACTH might be involved in Wnt-related depression-like behaviors. Many studies have shown that the VH had glutamatergic input to the paraventricular hypothalamic nucleus (PVN), while PVN is responsible for coordinating the regulation of hypothalamic–pituitary–adrenal axis.^55, 56^ Therefore the alteration of CORT and ACTH levels may be related with the PVN, which is regulated by the VH directly. However, the detailed mechanism still needs to be studied in the future. Furthermore, we found that knocking down Wnt2 or Wnt3 induced impaired Wnt/β-catenin signaling and deficits in neurogenesis. Previous studies found that GSK3β has been implicated in the regulation of mood-related behaviors and the treatment of antidepressants.^57, 58^ Our result suggested that selectively decreased Wnt2 and Wnt3 levels might be one of the mechanism underlying the abnormality of GSK3β activity in depression disorder. Wnt2 regulates progenitor proliferation and increases the number of dopaminergic neurons in the developing ventral midbrain.^38^ However, it is unclear what the role of Wnt2 in AHN is. Our results showed that knocking down Wnt2 impaired cell proliferation and decreased the number of newly generated neurons, which suggested that Wnt2 was also involved in AHN.

Second, we found that Wnt2 or Wnt3 was sufficient for buffering CRS-induced depression-like behaviors. Overexpression of Wnt2 by infusion of AAV-Wnt2 had an antidepressant effect under basal condition.^49^ However, the effect of overexpressing Wnt2 or Wnt3 on CRS-induced depression-like behaviors remains unknown. To the best of our knowledge, our results provided the first evidence showing that overexpressing Wnt2 or Wnt3 could produce an antidepressant effect under stress condition. Furthermore, we found that overexpression of Wnt2 or Wnt3 could rescue CRS-induced Wnt/β-catenin signaling and hippocampal neurogenesis deficits. Recently, ketamine, a fast-acting antidepressant, could inhibit the activity of GSK3β in the brain. Moreover, the inhibition of GSK3β activity is necessary for rapid antidepressant effect of ketamine, which suggested that GSK3β activity plays an important role in antidepressant.^57, 59^ To date, although the effects of reducing hippocampal neurogenesis on producing depressive behaviors are controversial, it is confirmed that AHN is essential for the therapeutic effects of fluoxetine in stressed mice.^60, 61, 62^ Therefore, the rescue of CRS-induced depression-like behaviors by overexpressing Wnt2 or Wnt3 might be mediated by regulating the GSK3β activity and/or hippocampal neurogenesis.

Third, we found that there is CREB-dependent positive feedback between Wnt2 and Wnt3. Our results showed that knockdown of Wnt2 could decrease, whereas overexpression of Wnt2 could increase Wnt3 levels and vice versa. These results suggested that the expression levels of Wnt2 and Wnt3 are associated with each other. Studies have reported that the Wnt signaling pathway could regulate the expression of calcium/calmodulin-dependent protein kinase IV (CaMKIV).^63^ CaMKIV activates the CREB/CRE transcriptional pathway via the phosphorylation of CREB.^64^ The transcription of Wnt2 and Wnt3 are CREB dependent.^47, 48^ However, there is no direct evidence showing that Wnt2 or Wnt3 could activate CREB. We found that overexpressing Wnt2 or Wnt3 in neurons could increase the level of p-CREB, and administration of CREB inhibitor could block the positive feedback effect between Wnt2 and Wnt3. The above data suggested that there is positive feedback between Wnt2 and Wnt3 that depends on the activation of CREB. For this reason, our current results are unable to clarify the role of wnt2 and wnt3 individually. Wnt3's effects have already been well-characterized in AHN,^9^ which is necessary for most antidepressants to have efficacy. Given this finding, in the future, experiments modulating levels of Wnt2 on the background of a Wnt3 deletion (and vice versa) will be necessary to resolve the function of each.

Finally, we found that Wnt2 and Wnt3 are necessary for antidepressant responses to fluoxetine treatment under stress condition. Voleti *et al.*^20^ reported that chronic electroconvulsive therapy could increase Fzd6, a Wnt receptor, and downregulate Dkk-2 expression, an inhibitor of Wnt/β-catenin signaling. Chronic fluoxetine administration has been reported to increase Wnt3a expression and reduce sFRP3 expression.^65, 66^ Okamoto *et al.*^49^ also reported that antidepressant treatments increase Wnt2 expression and activate the Wnt/GSK3β signaling under basal condition. However, whether Wnt2 mediates the effects of antidepressant treatment has not been demonstrated in the previous report. In our study, we found that chronic fluoxetine treatment could increase Wnt2 and Wnt3 levels under stress condition, suggesting that Wnt2 and Wnt3 may be involved in the antidepressant response to fluoxetine under stress condition. Studies have reported that Wnt2 and Wnt3 transcription are CREB dependent.^47, 48^ Fluoxetine treatment could increase the transcriptional activity of CREB.^67^ These studies suggested that fluoxetine might increase Wnt2 and Wnt3 levels by activating the transcriptional activity of CREB. Moreover, our study provided evidence that knockdown of Wnt2 or Wnt3 abolished the antidepressant effect of fluoxetine, suggesting that Wnt2 and Wnt3 are essential for mediating the antidepressant effects of fluoxetine under stress condition. In addition, we found that knockdown of Wnt2 could synchronously exacerbate the CRS-induced decrease in Wnt3 and block the fluoxetine-induced increase in Wnt3 and vice versa. This provided further confirmation that the expression profiles of Wnt2 and Wnt3 are associated with each other.

Taken together, our results demonstrated that Wnt2 or Wnt3 in the VH is necessary and sufficient for alleviating depression-like behaviors. Wnts are secreted glycoproteins that can not only act locally but may also have functions in other brain regions. Recent studies have reported that, besides the VH, Wnt2/3 are abundantly expressed in other brain regions, such as the striatum.^68, 69^ This suggests that the functions of Wnt2/3 in other brain regions also need to be considered in the future. Moreover, we found that Wnt2 and Wnt3 could activate CREB and that there is CREB-dependent positive feedback between Wnt2 and Wnt3. Another novel and significant finding of this study was that Wnt2 or Wnt3 is necessary for the actions of fluoxetine under stress condition. Our study provided novel insight into the relationship between Wnts and stress-induced depression-like behaviors. Importantly, the screening of small molecule compounds to increase levels of Wnt2 or Wnt3 could be a method for identifying potential new targets for interventions for depression.
