# Supplementary material for: The antidepressant roles of Wnt2 and Wnt3 in stress-induced depression-like behaviors
Source: Transl Psychiatry. 2016 Sep 13;6(9):e892–. doi: 10.1038/tp.2016.122 (PMC5048193; doi:10.1038/tp.2016.122)
Supplement: Supplementary Information [file tp2016122x1.doc]

**Supplementary information**

1. Supplementary material and methods
2. Supplementary references
3. Figure S1: The effect of CRS on depression- and anxiety-like behaviors, and body weight
4. Figure S2: Confirmation of hippocampus dissection by real-time PCR assay
5. Figure S3: Wnt2 and Wnt3 mRNA levels changes in the VH after a single restrained treatment
6. Figure S4: The specificity of some Wnt antibodies
7. Figure S5: The efficiency of siRNA lentivirus and the effect of Lenti-siWnt2 or Lenti-siWnt3 on anxiety-like behaviors
8. Figure S6: The effect of CRS on adult neurogenesis in the DH and VH respectively
9. Figure S7: The effect of Lenti-Wnt2 or Lenti-Wnt3 on anxiety-like behaviors
10. Table S1: Sequence of primers used for quantitative real-time PCR
11. Table S2: Animals used in different experiments

**Supplementary materials and methods**

**Protein extraction and Western blot**

Cytoplasmic and nuclear proteins were extracted using the Pierce Nuclear and Cytoplasmic extraction reagents (Thermo, # 78833), following the manual [^1^](#_ENREF_1). The ventral hippocampus (VH) and dorsal hippocampus (DH) were dissected on ice and then homogenized in ice-cold CER I (400 μl) for 15 s of vigorous vortexing. The homogenization was then incubated on ice for 10 min. Precooled CER II (22 μl) was added to the tube. After vigorous vortexing and incubation on ice for 1 min, the homogenization was centrifuged at 16,000 × g for 5 min at 4 °C and the supernatant collected as cytosolic protein. The precipitate was vibrated in ice-cold NER (200 μl) intensively for 15 s. The sample was placed on ice and vortexed for 15 s every 10 min, for 40 min. Then the precipitate was centrifuged at 16,000 × g for 5 min at 4 °C. The supernatant represented purified nuclear protein. The protein concentration was detected using the BCA protein assay. Then the proteins were used for western blot. The primary antibodies used in experiments are as follows: rabbit anti-phospho-GSK3β antibody (Cell Signaling Technology, #9336), rabbit anti-β-actin antibody (Sigma Aldrich, #A2066), rabbit anti-Wnt2 (Sigma Aldrich, #SAB2103591), rabbit anti-phospho-CREB antibody (Cell Signaling Technology, #9191), rabbit anti-CREB antibody (Cell Signaling Technology, #9197), rabbit anti-flag antibody (Thermo, #PA1-984B), rabbit anti-calnexin antibody (sigma, #C4731), rat anti-Wnt3a antibody (R&D, #MAB1324), mouse anti-β-catenin antibody (BD, #610154), mouse anti-Wnt3 antibody (Invitrogen, #1217179A), mouse anti-α-Tubulin antibody (sigma, #T5168), mouse anti-GSK3β antibody (BD, #610201), goat anti-LaminB antibody (santa, #sc-6216). Secondary antibodies: horseradish peroxidase (HRP)-conjugated rabbit anti-goat IgG antibody and horseradish peroxidase (HRP)-conjugated goat anti-mouse, rat or rabbit IgG antibodies were from Calbiochem. Immunoreactive protein bands were analysed by densitometry using Quantity one.

**Identification of antibody specificity**

Flag-tagged Wnt2, Wnt3 and Wnt3a were subcloned into the pCDNA3.1 expression vector (Invitrogen). All of the constructs were made using PCR and were confirmed by DNA sequencing. Cultured HEK293 cells were transfected with flag-tagged Wnt2/3/3a construct individually using Lipofectamine 2000 (Invitrogen). Flag and Wnt2/3/3a were detected respectively 72 h after transfection as shown in Figure S4. Western blots showed that the Wnt antibodies were specificity.

**Open field test**

Locomotor activity and anxiety-like behavior were measured in a square box (40 cm × 40 cm × 35 cm) within a dedicated testing rooom and illuminated at light intensities below aversive levels (20 lx). The open field was divided into central (20 cm × 20 cm) and peripheral areas. Mice were placed in the center of the field and allowed 10 min exploration. A videotracking system (Smart) was used to score the total distance traveled, a measure of locomotor activity and time spent in the central areas, a measure of anxiety-like behavior.

**Elevated plus maze**

The elevated plus maze was constructed of black stainless steel. The apparatus was raised to a height of 50 cm above the floor and consisted of two open arms (30 cm× 5 cm), two closed arms (30 cm× 5 cm) with high walls and a center platform (5 cm× 5 cm). The illumination level was 20 lx in the open arms and 5 lx in the closed arms. The test mouse was placed in the center platform facing an open arm and their behavior was recorded for 5 min. The percentage of time spent in open arms and percentage of entries into the open arms were used as a measure of anxiety-like behavior.

**Measurement of serum** **corticosterone and** **adrenocorticotropin hormone (ACTH) levels**

To determine the effects of Wnt2 or Wnt3 on corticosterone secretion and ACTH levels, mice were decapitated, and trunk blood from each mouse was collected immediately into tubes containing 7.5% EDTA. Samples were inverted gently to avoid coagulation and plasma was collected by centrifugation (1000 × g for 15 min at 4 °C) and stored at -80 °C for hormone assays. CRS mice were decapitated immediately after the last restraint stress (10:00 A.M.). No-CRS mice were also rapidly decapitated at the same time. Serum levels of corticosterone were determined using a corticosterone RIA Kit according to the manufacturer’s instructions (Beijing North Institute of Biotechnology). Serum levels of ACTH were measured using an ACTH RIA Kit according to the protocol offered by the company.


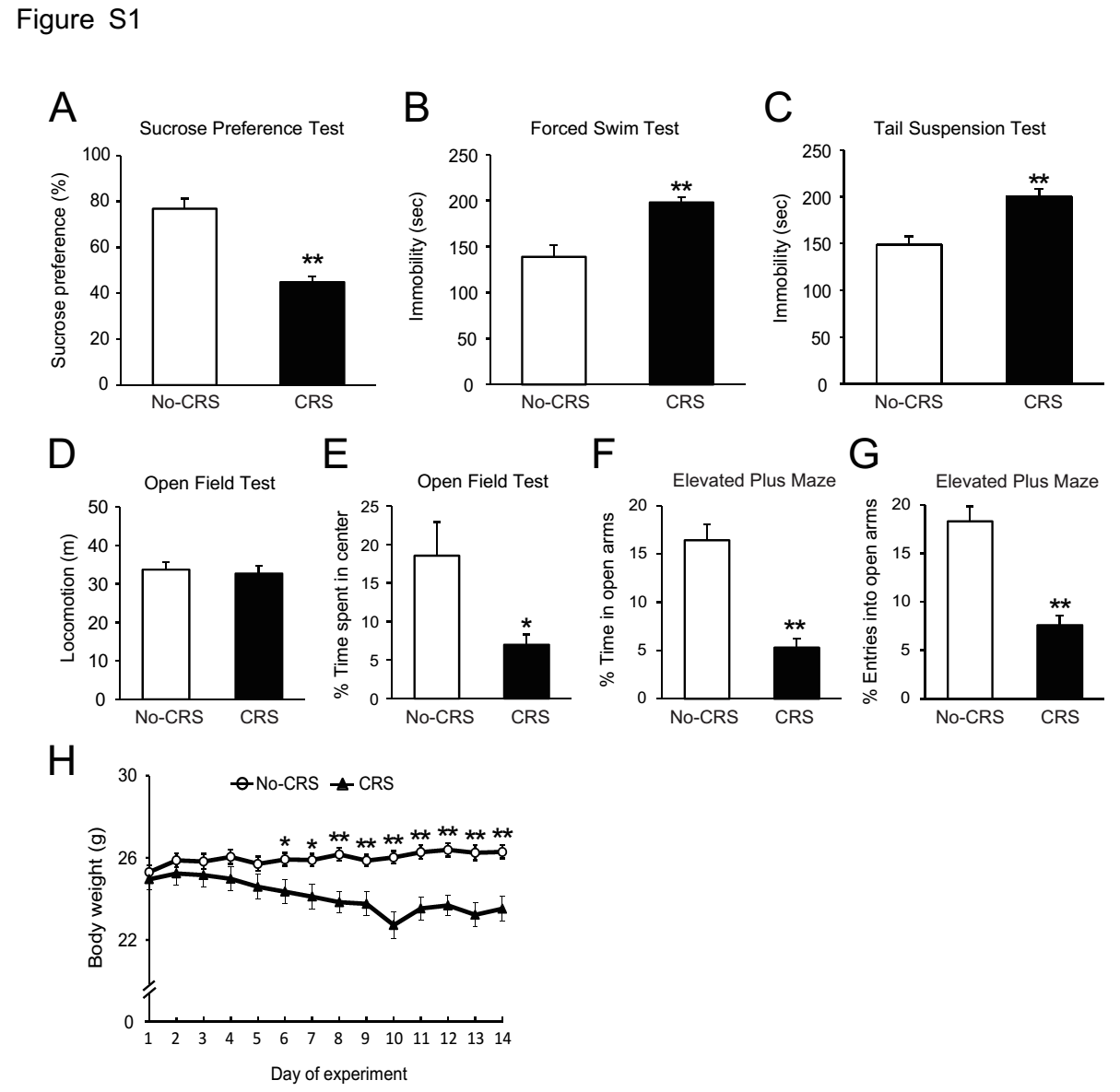


**Figure S1** The effect of CRS on depression- and anxiety-like behaviors, and body weight. (A) In the SPT, the CRS group showed decreased sucrose consumption compared with the No-CRS group. (B, C) In the FST and TST, the CRS group showed increased immobility time compared with the No-CRS group. (D) In the OFT, the CRS group showed no difference in locomotor activity compared with the No-CRS group. (E) In the OFT, the CRS group showed a decreased percentage of time spent in the center. (F, G) In the EPM, the CRS group showed a decreased percentage of time spent in the open arm (F) and percentage of entries into the open arms (G). (H) Daily body weight of mice exposed to 2 h of restraint daily for 14 consecutive days. n = 8 per group; **P* < 0.05, ***P* < 0.01 versus the No-CRS group. All values are denoted as the mean ± SEM.


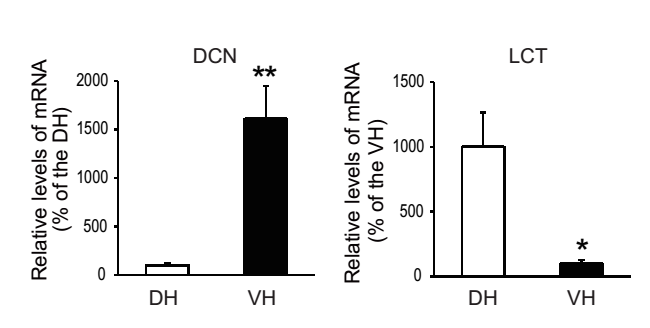


**Figure S2** Confirmation of hippocampus dissection by real-time PCR assay. Differential expression of Dcn and Lct in the VH and DH. n = 6 per group; **P* < 0.05, ***P* < 0.01. All values are denoted as the mean ± SEM.


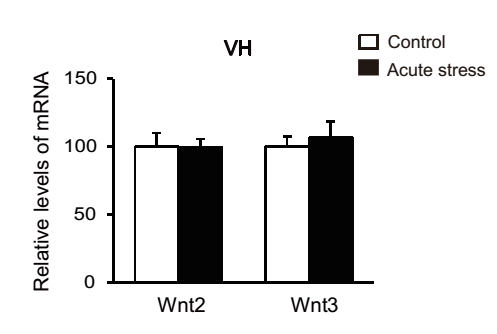


**Figure S3** Wnt2 and Wnt3 mRNA levels changes in the VH after a single restrained treatment. n = 10 per group. All values are denoted as the mean ± SEM.


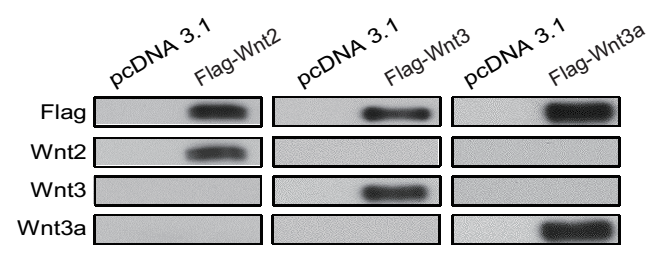


**Figure S4** The specificity of some Wnt antibodies.


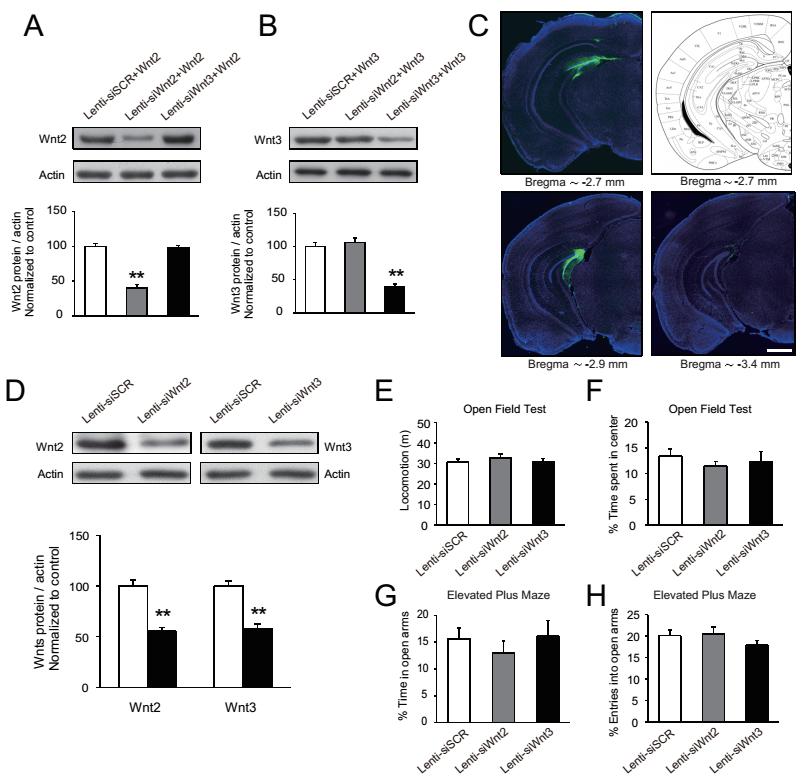


**Figure S5** The efficiency of siRNA lentivirus and the effect of Lenti-siWnt2 or Lenti-siWnt3 on anxiety-like behaviors. (A) Wnt2 expression in cultured HEK293 cells that overexpressing Wnt2 after siWnt2 or siWnt3 transfection. n = 6 per group; ***P* < 0.01 versus the Lenti-siSCR + Wnt2 group. (B) Wnt3 expression in cultured HEK293 cells that overexpressing Wnt3 after siWnt2 or siWnt3 transfection. n = 6 per group; ***P* < 0.01 versus the Lenti-siSCR + Wnt3 group. (C) Photomicrograph of the VH 5 weeks after lentivirus injection. The GFP fluorescence was used to determine the area to which the virus spread, and the GFP was found to be expressed within the dentate gyrus of the ventral hippocampus. The scale bar represents 800 µm. The upper right panel is a wider-field of view of the Atlas image, which represents the upper fluorescent immunostaining of GFP. (D) The levels of Wnt2 and Wnt3 in the VH, following Lenti-siSCR, Lenti-siWnt2 or Lenti-siWnt3 injection, were analyzed by western blot 5 weeks after injection. Lenti-siSCR group, n = 6, Lenti-siWnt2 and Lenti-siWnt3 groups, n=7; ***P* < 0.01 versus the Lenti-siSCR group. (E, F) In the OFT, Lenti-siWnt2 or Lenti-siWnt3 group showed no difference in the locomotor activity (E) and the percentage of time spent in the center (F) compared with Lenti-siSCR group. n = 10 per group. (G, H) In the EPM, Lenti-siWnt2 or Lenti-siWnt3 group showed no difference in the pertentage of time spent in the open arm (G) and percentage of entries into the open arms (H). n = 10 per group. All values are denoted as the mean ± SEM.


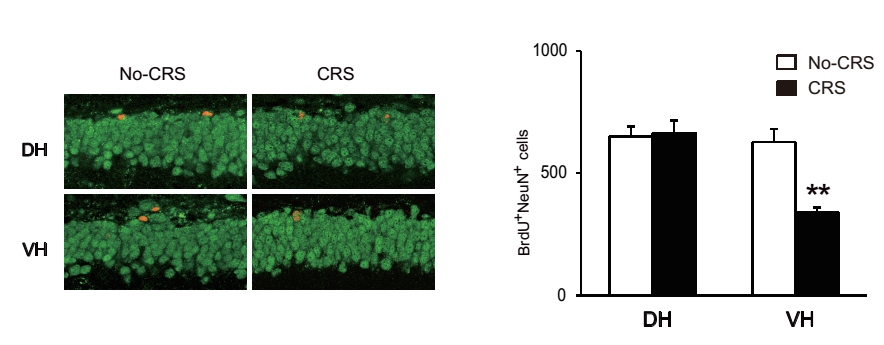


**Figure S6** The effect of CRS on adult neurogenesis in the DH and VH respectively. CRS could significantly decrease the numer of BrdU^+^NeuN^+^ cells in the VH but not DH. No-CRS: n = 4; CRS: n = 5; ***P* < 0.01 versus the No-CRS group. All values are denoted as the mean ± SEM.


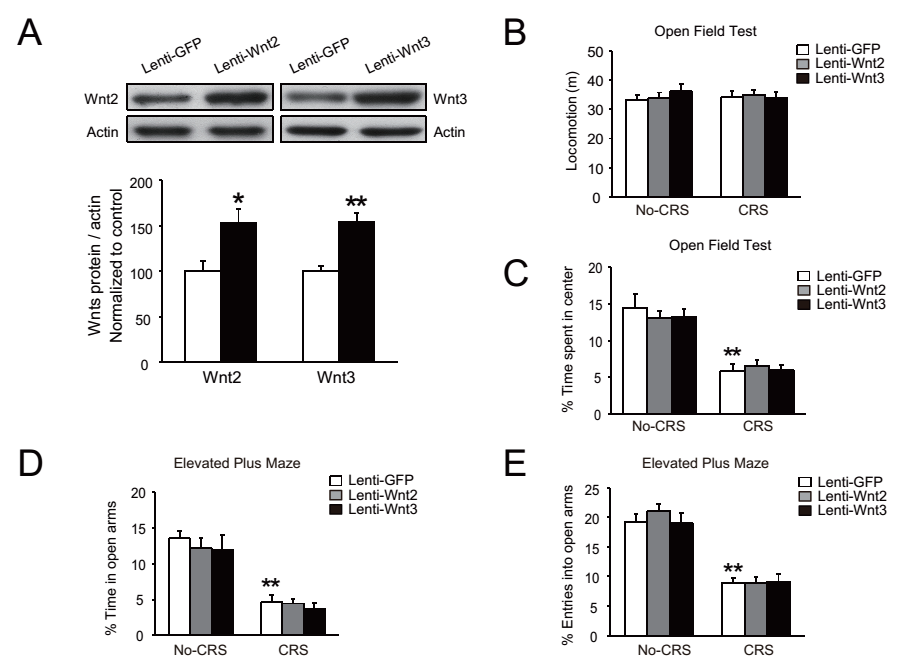


**Figure S7** The effect of Lenti-Wnt2 or Lenti-Wnt3 on anxiety-like behaviors. (A) Levels of Wnt2 or Wnt3 in the VH of Lenti-GFP, Lenti-Wnt2 or Lenti-Wnt3 treated mice were analyzed by western blot 5 weeks after injection. Lenti-GFP group, n = 5, Lenti-Wnt2 group, n = 6, Lenti-Wnt3 group, n= 7; **P* < 0.05, ***P* < 0.01 versus the Lenti-GFP group. (B) In the OFT, overexpressing Wnt2 or Wnt3 had no effects on the locomotor activity under basal or stress condition. (C) The effect of overexpressing Wnt2 or Wnt3 on the percentage of time spent in the center under basal or stress condition. n = 10 per group; ***P* < 0.01 versus the Lenti-GFP group. (D, E) In the EPM, the effect of overexpressing Wnt2 or Wnt3 on the percentage of time spent in the open arm (D) and percentage of entries into the open arms (E) under basal or stress condition. n = 10 per group;***P* < 0.01 versus the Lenti-GFP group. All values are denoted as the mean ± SEM.


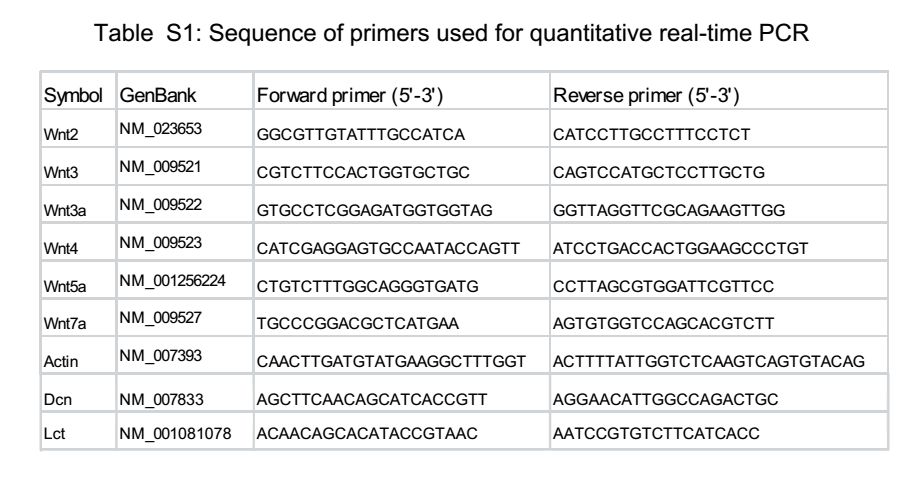


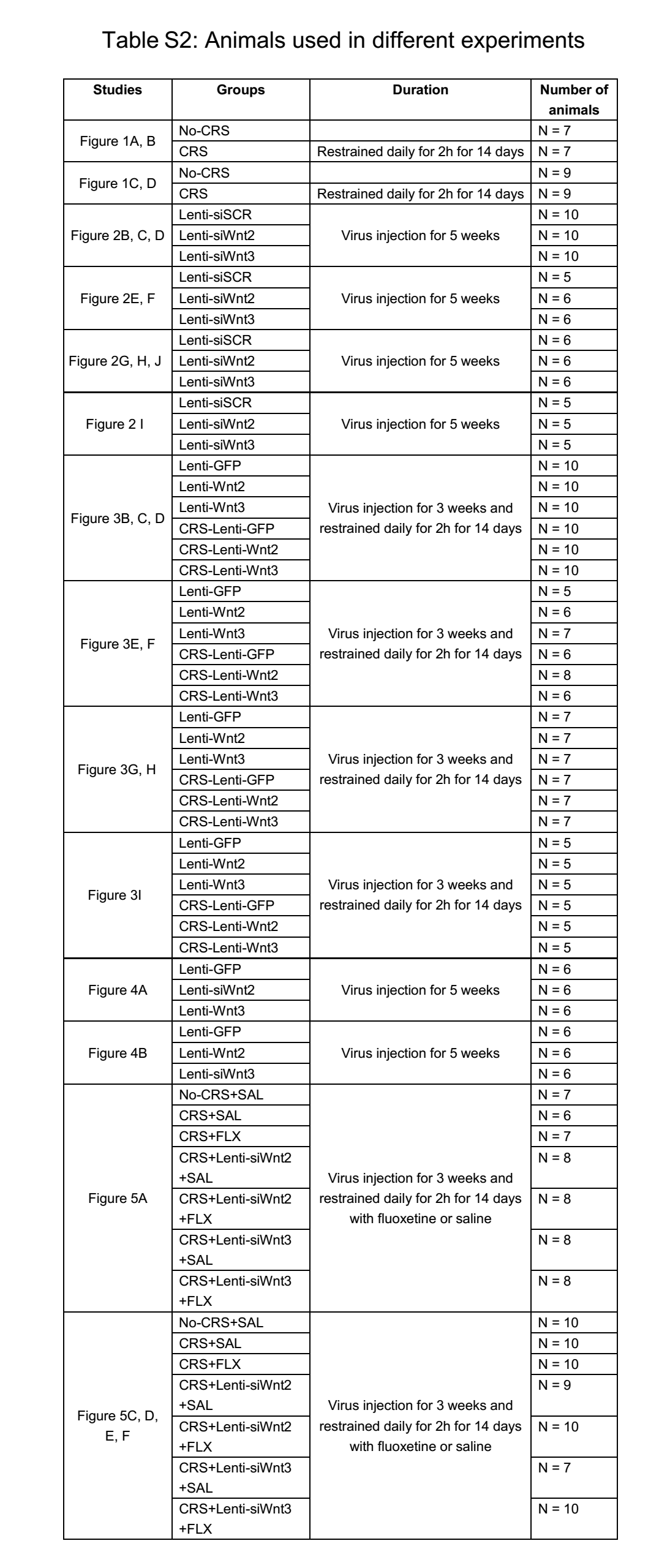


**Supplementary references**

1. Li B, Jie W, Huang L, Wei P, Li S, Luo *Z, et* al. Nuclear BK channels regulate gene expression via the control of nuclear calcium signaling*. Nature neuroscien*ce 2014**;** 17(**8**): 1055-1063.
